# Loeys‐Dietz Syndrome and Asthma: Pathophysiological Insights and Clinical Dilemmas

**DOI:** 10.1002/rcr2.70312

**Published:** 2025-08-13

**Authors:** Ella Brockwell‐Mole, John D. Blakey, Vidya V. Navaratnam

**Affiliations:** ^1^ Sir Charles Gairdner Hospital, Respiratory Medicine Perth Western Australia Australia; ^2^ Curtin University, Medical School Bentley Western Australia Australia; ^3^ Institute for Respiratory Health University of Western Australia Perth Western Australia Australia

**Keywords:** asthma, eosinophilia, Loeys‐Dietz syndrome, Oscillometry

## Abstract

A 23‐year‐old man presented with a life‐threatening asthma exacerbation following a respiratory infection. Although he had a childhood history of asthma, he had remained asymptomatic in adulthood and reported regular use of both vapes and marijuana. His medical history included Type 2 Loeys‐Dietz syndrome (LDS), requiring multiple vascular surgeries, rendering standard asthma therapies relatively contraindicated. Due to the severity of his condition, nebulised beta‐agonists were cautiously administered, and his beta‐blocker therapy was substituted. Imaging and forced oscillation technique (FOT) demonstrated severe obstructive airways disease. Marked and persistent eosinophilia was noted. While asthma is a recognised comorbidity in LDS, earlier recognition and intervention, such as inhaled corticosteroids, smoking cessation support, and judicious use of beta‐blockers, may have reduced the severity. Increased awareness of the prevalence and severity of asthma in LDS could support earlier diagnosis, preventive strategies and timely escalation of therapy, thereby reducing the risk of catastrophic respiratory events.

## Graphical abstract

We present a case of acute severe asthma in a young adult with Loeys‐Dietz syndrome, complicated by cardiovascular comorbidities. This case highlights the high prevalence and severity of asthma in LDS, the risks of delayed treatment and the importance of balancing bronchodilator therapy against vascular complications. Early recognition and preventive strategies are crucial to avoid life‐threatening deterioration in this population.

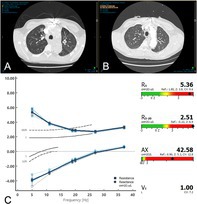

## 1 Introduction

Asthma is common globally, affecting millions of people of all ages. In the majority of individuals, the cause of asthma is unclear, and the amount of preventative therapy required to maintain control becomes apparent over time. Rare inherited diseases can shed light on the mechanisms of other more common conditions, but therapeutic dilemmas may also emerge in these multimorbid individuals.

## 2 Case Report

We present the case of a 23‐year‐old male who was admitted for an acute severe asthma exacerbation. Three weeks prior, he was diagnosed with respiratory syncytial virus (RSV) and developed progressive dyspnoea, fevers and malaise. On admission to the emergency department, he was in significant respiratory distress.

On examination, he was pale, tachypneic and had apparent respiratory fatigue. Auscultation revealed diffuse wheezing and decreased breath sounds. He was hypoxic on arrival, requiring 4 L of oxygen. The patient's medical history was notable for multiple vascular complications related to Type 2 Loeys‐Dietz syndrome (LDS), including Type A and B aortic dissections requiring aortic arch replacement and aortic valve replacement. Additionally, he had a history of pseudoaneurysms and rheumatic heart disease. He was advised to avoid beta‐agonists due to the risk of hypertension‐related vascular compromise. He exhibited arachnodactyly, hypermobile joints and pectus deformity. His past medical history also included childhood asthma, managed with salbutamol. He had no hospitalisations and no symptoms after age 11. Notably, the patient's social history indicated regular daily use of vaping products and bi‐weekly consumption of marijuana.

**FIGURE 1:**
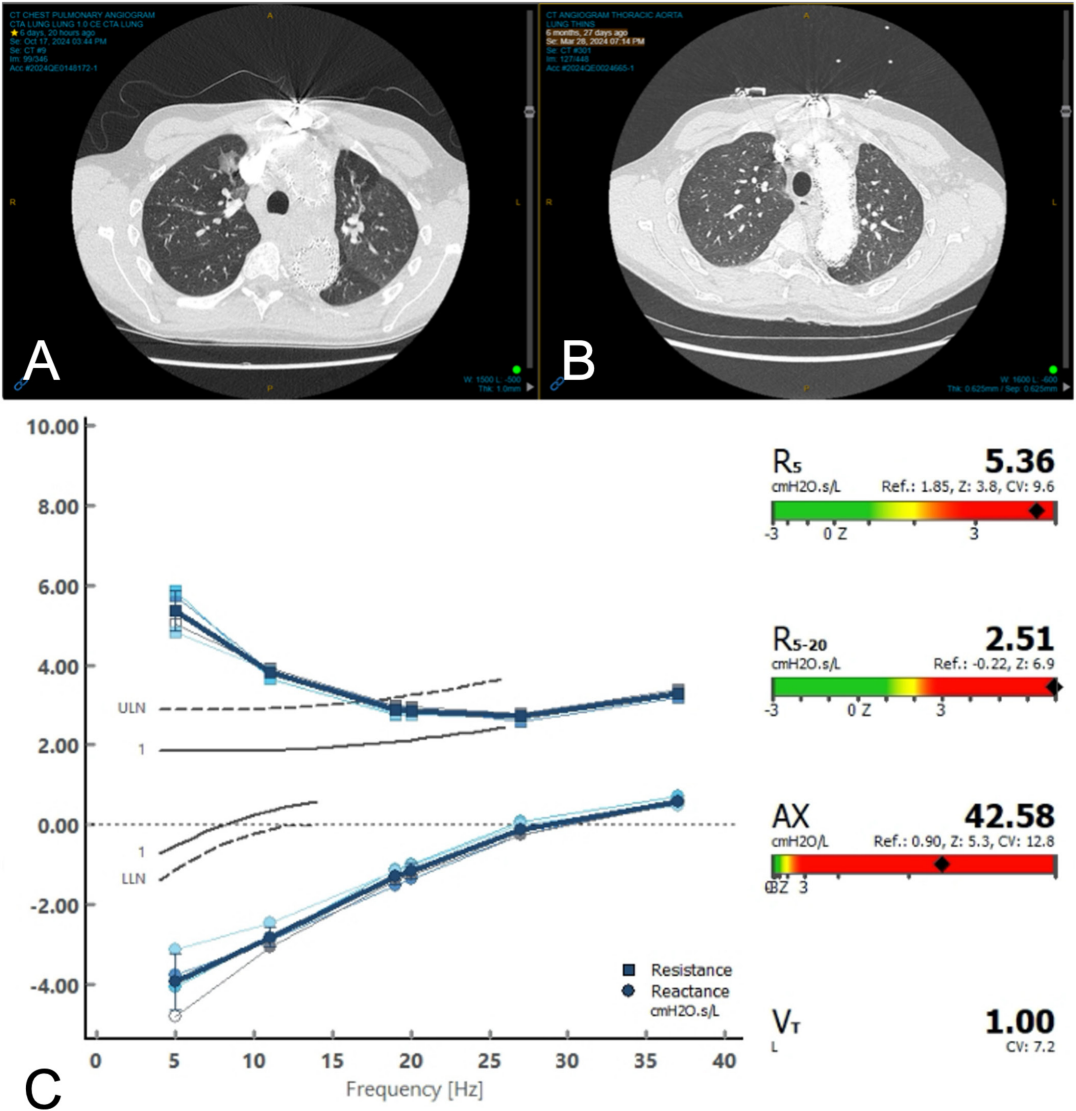
Diagnostic investigations at first presentation of adult‐onset asthma in a patient with Loeys‐dietz syndrome. (A) High‐resolution computed tomography (HRCT) of the chest during acute asthma exacerbation demonstrating bronchial wall thickening and mosaic attenuation consistent with small airway disease. (B) CT of the chest performed 6 months prior to presentation, in the absence of respiratory symptoms, showing persistent airway wall thickening. (C) Forced oscillation technique (FOT) measurements revealing elevated respiratory system resistance, supporting the diagnosis of small airway dysfunction.

Initial management consisted of inhaled muscarinic antagonists and systemic corticosteroids, providing some relief but with overall clinical deterioration. After multidisciplinary consultation involving Intensive Care, Emergency, Cardiothoracic and Respiratory teams, a decision was made to cautiously administer nebulised beta agonists. This intervention markedly improved his clinical status, albeit accompanied by induced tachycardia. He was closely monitored, and no complications ensued. Laboratory investigations revealed an eosinophil count of 1.29 × 10^9^/L. Computed tomography (CT) of the chest demonstrated airway wall thickening, secretions and mosaic attenuation. A scan performed earlier that year had shown similar signs, even in the absence of respiratory symptoms. See Figure 1A,B. Evaluation using forced oscillation technique (FOT) demonstrated elevated resistance at 5 Hz (R5) and increased R5–R20 difference, indicating small airway obstruction, in keeping with a diagnosis of asthma of higher severity and poorer control. See Figure 1C. Individuals with LDS are discouraged from physical activity, so this level of airflow limitation may not have been as apparent as it would have been in another individual.

For long‐term management, the patient's regular beta‐blocker was replaced with alternative antihypertensives; he was discharged with a regimen of preventive and rescue inhalers. Support for vaping and marijuana cessation was provided.

## 3 Discussion

Loeys‐Dietz syndrome (LDS) is an autosomal dominant connective tissue disorder characterised by a diverse array of systemic manifestations, including craniofacial, skeletal, cutaneous and vascular abnormalities [1, 2]. Initially defined in 2005, LDS is classified into five types based on mutations affecting the TGF‐β and SMAD pathways [2]. Genetic testing results for this case confirmed he was heterozygous for the TGF‐β2R mutation, confirming the presence of Type 2 LDS [2].

**FIGURE 2:**
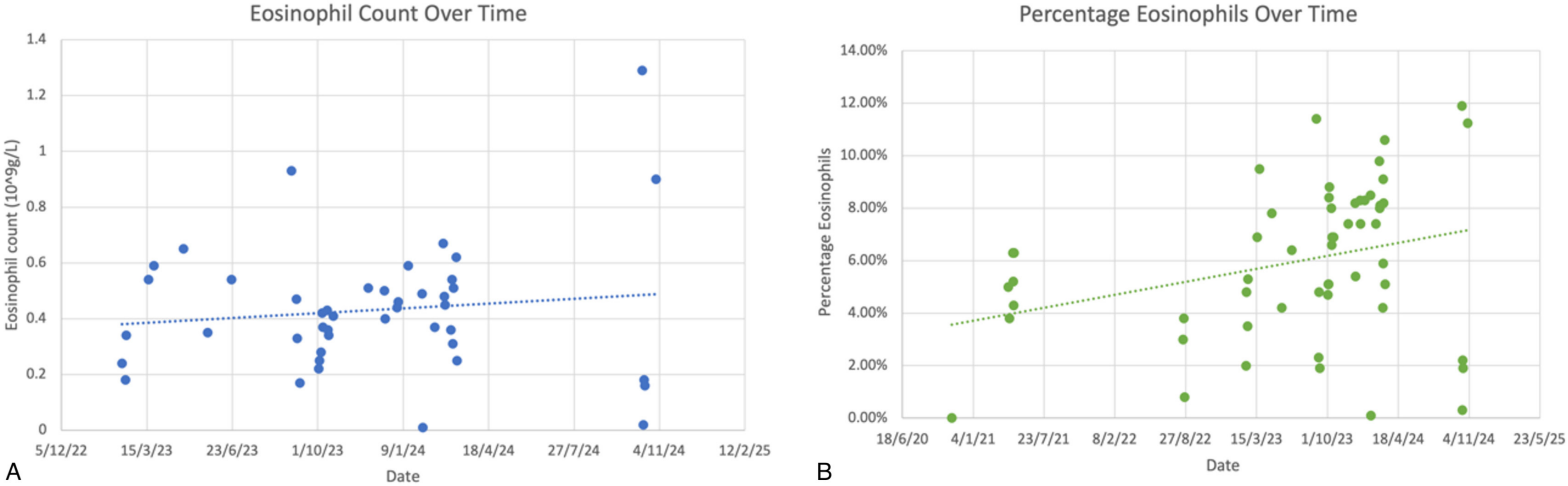
Longitudinal trends in peripheral blood eosinophils over a 3‐year period. (A) Absolute eosinophil count (×10^9^/L) plotted over time, demonstrating persistently elevated levels. (B) Percentage of eosinophils relative to total leukocyte count over time, highlighting sustained eosinophilia.

Asthma is very common in LDS, with case series publications reporting a prevalence of up to 50% [1]. LDS caused by mutations in TGF‐β1 and TGF‐β2 pathways appears particularly associated with type 2 helper T‐cell (Th2) driven allergic diseases, including asthma [1, 2]. Patients with LDS have been demonstrated to have significantly elevated eosinophil counts [2]. This patient's eosinophil counts over a 3‐year period are plotted below. See Figure 2. Dysregulated TGF‐β signalling drives eosinophilic inflammation and airway remodelling. In this case, the patient's persistent eosinophilia and severe airway disease may reflect a heightened Th2 immune response [3, 4]. The relationship between peripheral blood eosinophil count and asthma exacerbation risk is well described, as is their clinical significance for prediction of the efficiency of monoclonal antibody therapies [4]. The findings in this case of persistent airway wall thickening observed on CT in the absence of respiratory symptoms and elevated small airway resistance on FOT are suggestive of underlying obstructive small airways disease. In patients with vascular pathology like LDS, forced spirometry may be relatively contraindicated due to the risks of pressure‐related complications. FOT offers an effort‐independent alternative and can detect differences between inspiratory and expiratory resistance and reactance, making it useful for identifying dynamic airway collapse [5]. The presence of structural abnormalities on imaging, coupled with physiological evidence of peripheral airway disease, would align with what is expected in TGF‐β‐mediated airway remodelling.

The complexity of this patient's background contributed to delays in administering life‐saving treatment. While the risk of vascular compromise due to induced tachycardia is significant, the risk of deterioration from an acute asthma attack is even more pronounced. Recommendations for the management of asthma in LDS suggest that bronchodilators should be used conservatively and instead recommend routine use of inhaled corticosteroids. In addition, beta‐blockers should be reconsidered as antihypertensive therapy due to their predisposition to exacerbations, particularly β_1_‐antagonists [5]. In this case, the triggering event may also have been avoided with vaccination advocacy for common viruses (i.e., influenza and RSV).

Marijuana use worsens lung function and poses risks in predisposed patients. Given the evidence linking marijuana smoking to airway thickening and an increased risk of emphysema, it is crucial to emphasise the need for stronger discouragement in these individuals.

Increased awareness of the prevalence and severity of asthma in LDS may promote earlier recognition, preventive interventions and appropriate escalation of therapy, reducing the risk of catastrophic respiratory events.

## Author Contributions

E.B.M. drafted the initial version of the article; J.D.B. and V.V.N. revised the article.

## Consent

The authors declare that written informed consent was obtained for the publication of this manuscript and accompanying images using the consent form provided by the Journal.

## Conflicts of Interest

J.D.B. or his institution have received income for advisory or educational activities from manufacturers of asthma treatments: Astra Zeneca, Boehringer‐Ingelheim, Chiesi, GSK, Sanofi.

## Data Availability

The data that support the findings of this study are available on request from the corresponding author. The data are not publicly available due to privacy or ethical restrictions.
